# Heterologous production of active ribonuclease inhibitor in *Escherichia coli *by redox state control and chaperonin coexpression

**DOI:** 10.1186/1475-2859-10-65

**Published:** 2011-08-08

**Authors:** Juozas Šiurkus, Peter Neubauer

**Affiliations:** 1 Thermo Fisher Scientific (formerly Fermentas), V.Graiciuno 8, LT-02241 Vilnius, Lithuania; 2 Chair of Bioprocess Engineering, Department of Biotechnology, Technische Universität Berlin, Ackerstr. 71-76, D-13355 Berlin, Germany

## Abstract

**Background:**

Eukaryotic Ribonuclease inhibitor (RI), belonging to the RNH1 family, is distinguished by unique features - a high sensitivity to oxidation due to the large number of reduced cysteins and a high hydrophobicity, which made most production approaches so far unsuccessful or resulted in very low yields. In this work efficient *in vivo *folding of native RI in the *Escherichia coli *cytoplasm was obtained by external addition of a reducing agent in tandem with oxygen limitation and overproduction of a molecular chaperonin. After optimisation of the production conditions in the shake flask scale the process was scaled up to high cell densities by applying a glucose limited fed-batch procedure.

**Results:**

RI production in a T7 RNA polymerase based system results in accumulation of aggregated inactive product in inclusion bodies. Combination of addition of the reductant DTT, low production temperature and coexpression of the chaperonin GroELS resulted in high level production of approximately 25 mg g^-1 ^CDW active RI in *E. coli *ER2566 pET21b, corresponding to approximately 800 kU g^-1 ^cell wet weight. Further conditional screening under fed-batch-like conditions with the EnBase^® ^technology and scale up into the bioreactor scale resulted in an efficient high cell density glucose and oxygen limited fed-batch process with a final cell dry weight of 25 g L^-1 ^and a total RI yield of app. 625 mg L^-1 ^(volumetric activity of 80,000 kU L^-1^). The *E. coli *based production constructs showed a very high robustness. The recombinant culture maintained its productivity despite the combination of the toxic growth conditions, the substrate limited production mode in tandem with a high level expression of several recombinant proteins, the set of molecular chaperonins and the target protein (RI).

**Conclusions:**

High level production of active RI in *E. coli *in a T7 RNA polymerase expression system depends on the following factors: (i) addition of a reducing agent, (ii) low production temperature, (iii) oxygen limitation, and (iii) co-overexpression of the chaperonin GroELS. The study indicates the strength of applying fed-batch cultivation techniques for the efficient optimisation of production factors already at the screening stage for fast and straight forward bioprocess development even for target proteins which show a complex folding behaviour. In our case none of the approaches alone would have resulted in significant accumulation of active RI.

## Background

*E. coli *is a favorable host for recombinant heterologous protein production. The robustness of this microorganism, fast and simple cultivation, easy genetical manipulation, the enormous amounts of available physiological data and molecular biology tools are key reasons for its widespread use. Despite these positive general characteristics, protein aggregation and/or improper folding are major obstacles which often lead to reduced functional product yields. Many examples indicate the limited capacity of the natural *E. coli *protein folding machinery for a high level accumulation of heterlologous proteins with features wich are not usual for the hosts protein portfolio. Examples are proteins with multiple disulfide bonds, very large proteins, proteins with a high hydrophobicity, and proteins with natural glycosylation. However, target specific engineering approaches and process optimisation can lead to success. In the meanwhile there exists an extensive toolbox of elements which overcome natural limitations of the *E. coli *system, such as vectors for coexpression of rare tRNAs, chaperons and foldases, hosts and vectors with improved disulfide oxidation and isomerisation characteristics, newly designed secretion tools, and hosts for superior expression of membrane proteins [1-3]. Even tools for modulation the glycoylation pattern of proteins seems to become a future option [4,5].

Due to their therapeutic importance the production of disulfide stabilised proteins has been a major research focus in the past. This research gave the general impression that the cytoplasm of *E. coli *is a reducing compartment and thus the production of proteins with reduced cysteines should be granted. Hyperoxia and oxydation stress are important factors in connection with protein folding in Eukaryotes (e.g. see recent review by [6]), but have been largely neglected for recombinant protein production in *E. coli*. Although oxidative damage of proteins by carbonylation in *E. coli *has been extensively studied, it has been barely considered in connection with heterologous protein expression. One milestone publication in this aspect is the effect of the dissolved oxygen level on the carbonylation level and activity of human interferon γ [7].

In the present study we show at the example of a protein of the ribonuclease inhibitor (RI) family, that the redox conditions in the cytoplasm are an important target for process optimisation if the aim is the production of soluble and active product.

Proteic RIs are a family of highly conserved proteins. The conservation of the amino acid sequence between different hosts, such as pig, cow, rat, mouse, sheep and human is as high as nearly 70% [8]. All eukaryotic ribonuclease inhibitors share the following characteristics: (i) a high content of reduced cysteins (30-32 residues, 7% of total amino acids), and (ii) a core composed of 15-16 repeated hydrophobic leucine rich motives [8,9]. Due to these features recombinant production of eukaryotic ribonuclease inhibitors has been a challenge. Earlier production trials of human and porcine RI in *E. coli *resulted in low functional yields and major aggregation [10,11]. For example the yield of functional porcine RI in *E. coli *was as low as 10 mg L^-1 ^[10] and in *Sacharomyces cerevisiae *0.2 mg g^-1 ^wet weight [12]. Recently we reported production of soluble and functional RI as a fusion to maltose binding protein (MBP) in *E. coli *[13] with a relatively high yield of 39 mg g^-1 ^cell dry weight which could be realised in a medium-density fed-batch process with a final yield of app. 800 mg L^-1 ^of MBP-RI fusion protein, and correspondingly 425 mg L^-1 ^of RI. Similar amounts of functional RI per cell unit from N-terminal fusion proteins were recently obtained by Guo et al. [14], however the authors did not try to maximize the volumetric yield.

Fusion proteins have the disadvantage that an extra proteolytic processing step is needed to obtain the authentic RI, which would be a limitation for industrial scale production. Also, although the tagged protein was soluble, the ~40 kDa MBP fusion partner had a negative impact on the RI specific activity. Therefore in a next study we intended to directly produce almost authentic RI. Therefore we applied two strategies: (i) a cytoplasmic construct with an N-terminal His tag and (ii) a periplasmic construct with a secretion signal which would be removed during the transport of RI into the periplasm [15]. Surprisingly, we only succeeded in high yields of soluble and active RI with the cytoplasmic construct, and only if the reductant dithiothreitol (DTT) was added to the cultivation medium. The same production approach also improved the periplasmic yield of RI. However unexpectedly, the yield of active and soluble RI was higher with the cytoplasmic compared to the periplasmic expression constructs. After further comprehensive optimisation, including different vector constructs and expression principles, the best yield of 320 mg L^-1 ^of active His_6_-RI was obtained, by combination of the addition of DTT with a low cultivation temperature (22°C) and reduced aeration (pO_2 _close to zero) [15].

In the present study we aimed to further increase the cytoplasmic yield of native RI, i.e. without any tag, by applying the very strong routinely used T7 RNA polymerase controlled expression system. Surprisingly, the tested ER2566 (*E. coli *B-strain) pET21b constructs behaved totally different compared to the earlier tested constructs (*E. coli *K-12, plasmids with a *lac*-derived promoter). DTT did not provide a positive effect on the yield of functional RI, possibly due to the imbalance between very strong protein synthesis and slow folding. The problem was solved by co-overexpression of the GroELS chaperonine. The yield of RI finally could be maximized by combining the sequential induction of GroELS and RI with a delayed addition of DTT and the maintenance or a low level of dissolved oxygen. This process strategy also was successful in a bioreactor under fed-batch process conditions with a final cell dry weight of 25 g L^-1 ^and a volumetric yield of 625 mg L^-1 ^of soluble and highly active RI.

## Results

**Figure 1:**
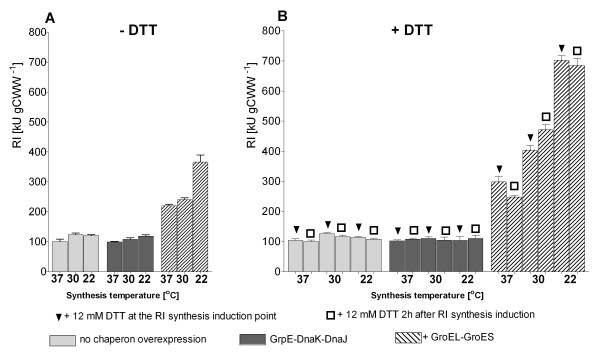
**RI protein activities in kilo units per gram of wet cell weight [kU (gCWW)^-1^] after shake flask batch cultivation of *E. coli *ER2566 pET21bRI or with additional chaperone co-overexpression using the pKJE7 (DnaK/DnaJ/GrpE) or pGro7 *(*GroELS) vectors at 22, 30 or 37°C in glucose-MSM without DTT (A) or with addition of DTT (B)**. Shake flask cultures were performed in glucose MSM at 37°C until an OD_600 _of 1 to 1.5. Chaperons were induced with 0.4 g L^-1 ^of arabinose 2 hours before RI induction (0.2 mM IPTG) at 37°C. The temperature was set at the time of RI induction to 22, 30 or 37°C, respectively. DTT (12 mM final concentration) was added at the time of RI induction (triangles) or 2 hours later (squares). Samples were collected 4 hours after RI induction. Standard deviations represent 2 independent cultivations and 3 assays.

**Figure 2:**
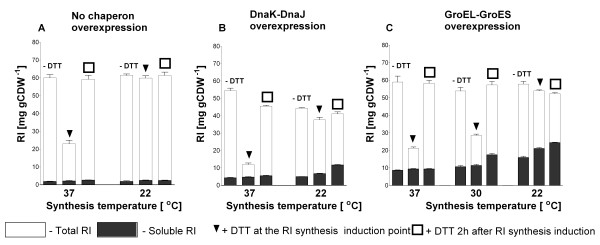
**Total (white bars) and soluble (black bars) RI amounts per cell unit [mg (g CDW)^-1^] after 4 hours of batch production in shake flask batch cultures of *E. coli *ER2566 pET21bRI (A) or with additional chaperone co-overexpression using the pKJE7 (DnaK/DnaJ/GrpE) (B) or *pGro7 *(GroELS) *(C) vectors *at 22, 30 or 37°C**. Cultures were performed in glucose-MSM medium with addition of 12 mM DTT at the time of RI induction (squares), 2 hours after RI induction (triangles), or no addition of DTT (control). Induction of GroELS was performed with 0.4 g L^-1 ^of arabinose 2 hours before RI induction (with 0.2 mM IPTG). All cultures were initially started at 37°C and shifted to the respective temperature at the time of RI induction. Standard deviations represent 3 assays.

In the present study we were interested to further develop the RI process for cytoplasmic production. Therefore we selected the widely used strong T7 RNA polymerase based expression system based on the vector pET21b and the *E. coli *B strain ER2566 as a production host. In the first set of production trails we applied supplementation with DTT and low production temperature, as those have been key parameters for active RI production in our previous approach [15]. Surprisingly, contrary to our expectations, DTT did not positively affect soluble accumulation, nor resulted in any higher RI activity in shake flask cultures of *E. coli *ER2566 pET21bRI, independent from the postinduction temperature and DTT addition time (Figures 1 and 2, also see SDS-Page gels in Additional file 1). Despite, a large amount of RI accumulated in inclusion bodies (Additional file 1).

A major difference between the previous and the actual expression systems is the strength of transcription. Previously we used a *lac *derived promoter, but here we used the T7 RNA polymerase based promoter system. The new system supports a very high rate of product synthesis corresponding to more than 60 mg gCDW^-1 ^(Figure 2 and Additional file 1) even at the low production temperature. Thus we assumed that the aggregation of RI could be due to the overloading of the cellular folding machinery by the hydrophobic and slowly folding RI. To test this hypothesis we coexpressed the chaperon systems DnaKJE and GroELS in tandem with RI.

While co-overexpression of DnaKJE did not provide any significant improvement in our case, coexpression of GroELS resulted in an increased RI solubility (Figure 2, Additional files 2 and 3) and activity (Figure 1). This positive effect was stronger at a lower postinduction temperature, but the best yield was obtained when DTT was added. The combination of GroELS and DTT resulted in 30-55% improvement of the accumulation of soluble RI (Figure 2 and Additional file 3) and a 1.5 to 2 fold increase in RI activity (Figure 1) compared to the trials without DTT. The best result was obtained when DTT was added two hours after RI induction in combination with a lower postinduction temperature of 30 or 22°C (Figures 1 and 2).

Remarkably, although the highest volumetric activity was obtained with the delayed addition of DTT, the highest activity per cell was obtained when DTT was added at the time of RI induction. In comparison to the processes without DTT the RI activity per cell increased by 90% when DTT was added at the time of RI induction, but only by 70% in the case of delayed DTT addition (Figure 1).

Interestingly, the combination of DTT and DnaKJE overproduction did only increase the solubility of RI, but the product was principally inactive (Figures 1, 2 and Additional file 2).

In conclusion, (i) the highest activity of RI per cell unit was obtained with a postinduction temperature of 22°C co-expression of GroELS and addition of DTT. (ii) The delayed DTT addition was preferred in the next set of experiments, as it seemed to be less detrimental to the culture viability.

### Fed-batch process in shake flasks

As previously, prior to the bioreactor experiments the fed-batch procedure was basically tested and optimised in shake flasks by applying the EnBase cultivation technique [16]. RI synthesis was induced 2 hours after induction of GroELS by addition of 0.2 mM IPTG, at the fed-batch cultivation mode at OD_600 _≈ 5 (μ ≈ 0.22 h^-1^) or OD_600 _≈ 11 (μ ≈ 0.10 h^-1^), respectively. Analogical to the batch processes, the cultivation medium was supplemented with DTT at the time of RI induction or 2 hours after induction. RI production was performed at 22°C for 4 hours. The effect of DTT on the production of RI and during co-expression of GroELS was evaluated by comparing the results to a culture without DTT addition.

**Figure 3:**
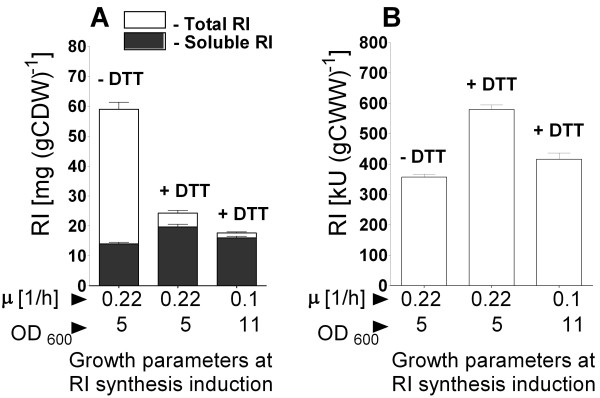
**RI protein yields after shake flask EnBase fed-batch cultivation of *E. coli *ER2566 pET21bRI *pGro7 *without DTT (-DTT) or with addition of 12 mM of DTT 2 hours after induction of RI (+DTT)**. Total (white bars) and soluble (black bars) RI amounts in milligrams per gram of dry cell weight [mg (g CDW)^-1^] (A) and RI activities in normalised crude extracts in kilo units per gram of wet cell weight [kU (g CWW)^-1^] (B) 4 hours after RI induction. The amounts of target protein in (A) were determined from scanned SDS-PAGE gel images as described in material and Methods. Standard deviations represent RI amounts from 3 assays.

Interestingly, independent from the cell density at the time of RI induction and in difference to the batch shake flask cultures described above, DTT addition at the time of RI induction had a negative effect on the accumulation of RI, even at the low synthesis temperature (results not shown). In contrast, delayed DTT addition was better, but still the total amount of RI decreased to about 50% in comparison to the fed-batch process without DTT (Figure 3, Additional file 4). However, despite the negative effect of DTT on the total amount of RI per cell (soluble and insoluble fractions), the yield of soluble and active RI per cell was doubled and the final volumetric activity was even five-fold improved compared to the previous batch experiments. Interestingly, the total amount of RI was higher at early induction (OD_600 _≈ 5) compared to late induction (OD_600 _≈ 11), indicating that the balance of synthesis rate and folding is an important optimisation parameter (Figure 3).

From this part we conclude that the most appropriate fed-batch growth conditions for RI production with GroELS and DTT would be to perform the induction of RI at a specific growth rate μ between 0.1 and 0.22 h^-1^. Preferably, DTT should be added 1 to 2 hours after RI induction.

### Bioreactor process

By taking the previous results into account, the batch and fed-batch RI production processes in the stirred bioreactor with the presence of DTT and GroELS overexpression were designed. Additionally, as a reference culture, also an RI production process without DTT was performed.

**Figure 4:**
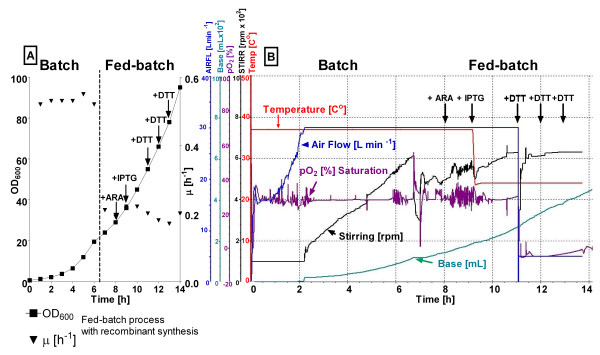
**Cell growth (A) and bioreactor parameters (B) of *E. coli *ER2566 pET21bRI *pGro7 *during fed-batch RI production with exponential glucose feeding and repeated DTT addition in a 10 L bioreactor**.

RI induction in a glucose limited fed-batch under substrate limitation in the stirred bioreactor cultures was performed at optical densities of app. 20 and 38. It was decided to maintain the specific growth rate in all fed-batch cultures at μ ≈ 0.22 ± 0.03 h^-1 ^by an exponential addition rate of the glucose feed solution. As a control also a batch culture was performed with induction of RI at an OD_600 _of 5 (μ ≈ 0.5 h^-1^). In all processes GroELS overexpression was induced 2 hours before induction of the target protein at 37°C (Figure 4) and the temperature was decreased to 22°C at the time of RI induction. Interestingly, in difference to our previously published fed-batch process with the lac-derived promoter system and the *E. coli *K-12 strain [15], in the current cultivations with coexpression of GroELS, the growth of the cultures was not completely inhibited by the addition of DTT, but the cells grew still with μ ≈ 0.15 h^-1 ^until the end (Figure 4).

By taking the previous study into account, the bioreactor cultivation medium was supplemented with DTT at two different modes; (i) a single DTT pulse addition starting 2 hours after RI induction and (ii) repeated DTT pulses, analogical starting 2 hours after RI synthesis induction. In all bioreactor processes, the air flow was reduced at the DTT addition point, from 30 to 3 - 5 L min^-1 ^to maintain the dissolved oxygen concentration in the cultivation medium close to 0%. The stirrer speed was kept the same as before induction (Figure 4).

**Figure 5:**
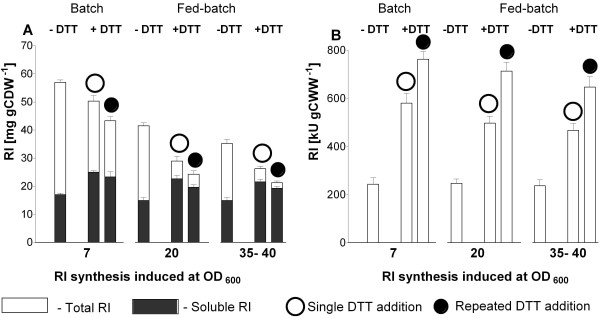
**RI amounts and activities normalized per cell amount from samples of batch and fed-batch bioreactor cultivations without and with addition of DTT that was either added once (open circles) or repeatedly (one pulse per hour, filled circles)**. The first addition of DTT was performed always two hour after induction of RI synthesis. A: Total (white bars) and soluble (black bars) RI amount as mg per g cell dry weight [mg (g CDW)^-1^]. B: RI activities in normalised crude extracts, in kilo units per gram of wet cell weight [kU (g CWW)^-1^]. The data are from 3 independent assays.

In agreement with the results from the small-scale study, also in the bioreactor fed-batch processes DTT decreased the accumulation of total RI by 30 to 40% compared to the processes without DTT (Figure 5, Additional file 5). However, despite the negative effect of DTT on the total yield of RI per cell, independently from cultivation mode, DTT improved the soluble amount of RI by 30 to 35%. The RI activity in cultures without DTT was very poor, but a 3.2 - 3.9 fold overall improvement of the activity per cell was obtained with repeated DTT addition compared to the processes without DTT. Interestingly, in contrast to the previously described *E. coli *K-12 process also a single DTT addition caused a good improvement of the RI activity with the new construct (2.2-2.8 fold overall improvement, Figure 5). This was surprising, as the DTT oxidation pattern in this process was similar as in the previously described process of *E. coli *RV308 K-12 [15]. After 3 hours of RI production at the micro-aerobic conditions only 40 to 50% of reduced DTT was detected in the bioreactor medium, and 85 to 90% in a shake flasks process, respectively.

Like in the shake flaks the highest amount of RI per cell unit (≈ 60 mg gCDW^-1^) was achieved in the batch bioreactor without DTT (Figure 5, Additional file 5). The RI amount per cell was approximately 15 - 30% lower during fed-batch operations, depending on the cell density at the synthesis induction point (Figure 5, Additional file 5).

Although the RI activity per cell unit was slightly lower in the fed-batch cultures compared to the batch cultures, the volumetric yield of active RI was much higher, especially when RI synthesis was induced at higher OD_600_. In this case final optical densities of 90 to 95 were reached, corresponding to 23 to 25 g cell dry weight per litre. The fed-batch process with repeated DTT addition yielded 625 mg L^-1 ^of RI corresponding to a volumetric activity of ~80,000 kU L^-1^.

## Discussion

In this work we established an efficient recombinant process for Ribonuclease inhibitor (RI) production, resulting in 625 mg L^-1 ^of active product with the authentic amino acid sequence. Previously we had improved cytoplasmic and periplasmic folding by either screening for a functioning fusion partner [13] or by controlling the redox situation by external addition of DTT [15] in the *E. coli *RV308 K-12 strain.

Surprisingly, the earlier published approach for cytoplasmic expression of authentic RI only worked in the *E. coli *K-12 system with a P*_lac _*promoter, but not in the strong T7 RNA polymerase based expression platform. In the T7 RNA polymerase system all product aggregated, possibly due to the high synthesis rate of RI and thus it outcompetes cellular folding factors. This hypothesis seems to be confirmed by the significant improvement of active RI by co-production of the GroELS chaperonins.

Our attempts to produce RI with chaperons also gave interesting and unexpected results, once more demonstrating the unpredictable effects of chaperon coproduction on the soluble accumulation of target proteins. In our case the most promising DnaKJE chaperone system only resulted in a improvement of RI folding, reflected by increase in the solubility of RI, but the product showed no activity. In contrast, co-production of GroELS had a large positive effect on the soluble accumulation and activity of RI. The best results were achieved at a low production temperature (see Results section Figures 1, 2). Although GroELS co-production has been successfully applied for many recombinant proteins (see recent review by Kolaj et al. [17]), our success was not granted, because there are many reports where GroELS overproduction failed to solubilise or to correctly fold proteins of interest [18-21]. The DnaKJE chaperone system is acting in the early stages of the folding pathway. Thus it possibly stabilizes nascent RI molecules by binding to the hydrophobic patches that stipulates solubility increase, but cannot facilitate complete formation of the native RI horse shoe structure because this would require additional more sophisticated folding. Interestingly, a closer look to the gels shows that DnaK is barely aggregating in tandem with RI, but DnaJ follows RI into the insoluble protein fraction, meaning that DnaJ and RI interact very strongly (Additional file 2). Possibly DnaJ remains bound also in the soluble protein fraction and prevents RI interaction with substrate during the activity assay.

The maximum RI activity and amount in the soluble fraction was achieved in all RI/GroELS co-production experiments at a low post-induction temperature, which fits well with the hypothesis that the cell machinery is easily overloaded by a high production of RI. It may be possible that the increase of solubility and activity of RI, having a size of 49 kD, was obtained by full encapsulation of the target protein within the GroEL barrel, which was stronger expressed in all cases compared to RI (see Figures 3, 4 and 7). Such a shielding RI from the environment may be advantageous to protect it from inactivation by oxidation and may be an explanation for the higher robustness of the GroELS coexpression process for the procedure of when and how DTT is added compared to the previous process where RI was simply expressed in the cytoplasm of *E. coli*.

Interestingly, in all shake flaks batch production experiments, the tandem of RI synthesis induction and DTT addition had a negative effect on the total amount of RI, especially when the production was carried out at higher temperatures. In our opinion, the combination of DTT toxicity, strong RI induction and high production temperature was a strong physiological stress, which drastically diminished cellular resources required for high level RI accumulation. For many other examples such impact of strong induction on cellular growth and recombinant production has been described before. In case of aggregation of the target protein a heat shock like response is induced which leads to accumulation of chaperones and proteases [22-24], changes in cellular respiration [25,26] and even ribosome destruction and loss of viability [27,28]. Interestingly however, somehow the cells in our case were capable to cope with the physiological stress. They maintain RI productivity when the temperature was decreased to 22°C and/or DTT was added only two hours after induction of RI.

Despite its growth inhibiting effect DTT had a positive effect on the solubility and activity of RI during production in shake flasks with GroELS coproduction, possibly by preventing oxidation of cysteins. These data are in good agreement with our previous data of RI production in the *E. coli *K-12 strain, were the dependency of the RI activity on the DTT concentration was demonstrated [15].

Before development of a high cell density bioreactor-scale fed-batch process it was important to test productivity and target protein folding capabilities of the ER2566 pET21bRI pGro7 construct under substrate limitation. As previously [13], we applied the EnBase fed-batch technique in parallel shake flask trials. We considered that strong co-overexpression of the target protein and the GroELS chaperonin, as well as other factors connected to the recombinant vectors, e.g. expression of the two antibiotic resistance genes, could withdraw critical amount of energy recourses leading to a loss of production capacity under glucose limited cultivation conditions, especially in the presence of toxic DTT. According to this expectation the combination of substrate limitation and DTT decreased the total amount of RI, but the amount of soluble and active RI was even slightly increased. This was also confirmed in bioreactor experiments which were performed by the same principle but at much higher cell densities.

Interestingly, in contrary to the previous study, in EnBase cultures even after a single pulse of DTT, chaperon-mediated RI folding resulted in a twofold higher RI activity compared to the reference process without DTT. However in the bioreactor, despite applying a very low aeration rate, DTT was fast oxidised and thus repeated addition of DTT was absolutely necessary for obtaining a high final product concentration, like in our previous study [15]. However, remarkably the negative effect of DTT oxidation on the folding of RI was not as strong as in the earlier production system, possibly by the shielding effect of GroELS.

In difference to the earlier published process with the K-12 strain and the *lac *promoter system, the cell growth showed a high robustness of the ER2566 pET21bRI pGro7 clone to the process conditions. After induction cell growth did not cease, but the culture was still growing with a specific growth rate of μ≈0.15 h^-1 ^until the end of the cultivation and was completely consuming the carbon source. This indicates metabolic activity and recombinant productivity at the very high cell densities even after repeated pulses of DTT and a final DTT concentration over 3.5 g L^-1^. We consider, that co-overexpression of GroELS stipulated such cell robustness against very harsh conditions by saving cellular host proteins which were affected by the high DTT concentrations.

## Conclusions

In this work a unique production strategy for RI was established which is based on co-overexpression of GroELS chaperonins, low production temperature and maintenance of reducing conditions. GroELS possibly shields the very slowly folding RI from the environment and thus prevents aggregation of this hydrophobic protein. Also the reducing environment in the GroEL barrel may avoid oxidation of the cysteins of RI. We believe that our strategies may be also important for the folding of other slow folding aggregation-prone proteins. Interestingly, coexpression of GroELS makes the cells more resistant to the toxicity of DTT. This is an interesting aspect that needs further functional investigation.

## Methods

### Expression strain preparation

The *E. coli *B strain ER2566 (New England Biolabs) was transformed with the plasmid pET21b-RI and was plated on LB agar with ampicillin (100 μg mL^-1^). The expression strain E. coli ER2566 pET21b-RI was co-transformed with the vectors pGro7 and pKJE7 (Takara Bio Inc) respectively, carrying the genes for the GroEL-GroES and DnaK-DnaJ chaperone systems. Transformants with both plasmids were plated on LB agar containing ampicillin (100 μg mL^-1^) and chloramphenicol (30 μg mL^-1^). Both transformations were based on the calcium temperature shock method. Glycerol cell stocks were produced after 8 h of cultivation of the transformants in liquid LB medium with the required antibiotics at 37°C and 220 rpm. A 50% sterile glycerol solution was used to produce 25% glycerol stock cell stocks which were aliquoted in Eppendorf tubes and stored at -70°C.

### Cultivation media

Transformations and plasmid propagations were performed on solid and liquid LB medium containing Bacto-Tryptone (10 g L^-1^), Bacto-yeast extract (5 g L^-1^), NaCl (10 g L^-1^), and for solid medium 15 g L^-1 ^bacto agar, as well as the required antibiotics. Fed-batch and batch cultivations were performed in glucose-based mineral salt medium (MSM) with the following composition (per litre): Na_2_SO_4 _2 g, (NH_4_)_2_SO_4 _2.68 g, NH_4_Cl 0.5 g, KHPO_4 _14.6 g, NaH_2_PO_4 _× H_2_O 3.6 g, (NH_4_)_2_-H-citrate 1.0 g, and glucose 10 to 15 g. NaOH (40%) was used to adjust pH to 7.0 prior to the heat sterilisation. Additionally, before cultivation the mineral salt medium was supplemented with the following sterile solutions: 3 mL L^-1 ^of (1M) MgSO_4 _and 2 mL L^-1 ^of trace element solution with the following composition (per litre): CaCl_2 _× _2_H_2_O 0.5 g, ZnSO_4 _× 7H_2_O 0.18 g, MnSO_4 _× H_2_O 0.1 g, Na_2_-EDTA 20.1 g, FeCl_3 _× 6H_2_O 16.7 g, CuSO_4 _× 5H_2_O 0.16 g, CoCl_2 _× 6H_2_O 0.18 g; as well as 100 μL L^-1 ^of thiamine hydrochloride (1 M), 1 mL L^-1 ^of ampicilin (100 mg mL^-1^) and 1 mL L^-1 ^of chrolamphenicol (30 mg mL^-1^). The feeding solution for fed-batch cultivations was based on fully formulated MSM with the required antibiotics and 550 g L^-1 ^of glucose.

### Batch mode cultivations and recombinant protein synthesis in shake flasks

The inoculums for batch protein production in the shake flasks were prepared by overnight cultivation of the selected clone in 500 mL shake flaks with 50 ml of MSM medium containing 10 g L^-1 ^of glucose at 37°C. For protein production 2 mL of the corresponding inoculum culture was transferred to fresh mineral salt medium containing 10 g L^-1 ^of glucose to a final volume of 200 mL in 1L baffled Erlenmeyer shake flasks. Cultures were cultivated at 37°C and 220 rpm until they reached the chaperon over-expression induction point, corresponding to a cell density of OD_600_≈ 0.5 ± 0.05 (μ = 0.42 ± 0.05 h^-1^). Induction was performed with of 0.4 g L^-1 ^of arabinose. RI was induced 2 hours after chaperon induction with 0.2 mM IPTG. The reducing agent dithiothreitol (DTT) was added to expression cultures as a dry powder to the cultivation medium at the RI induction point or 2 hours after RI induction to achieve a final concentration of 12 mM. The temperature was changed at the RI induction point to 22, 30, or 37°C and the culture was continued for 4 hours at 220 rpm.

### Fed-batch mode cultivations and recombinant protein synthesis in shake flasks

The EnBase^® ^technology based fed-batch shake flask cultivations were performed in 1 L baffled Erlenmeyer flasks in 200 mL of MSM as described before by Siurkus et al. [13]. In all experiments the cells were cultivated at the substrate limited mode, generated with 12 AGU L^-1 ^of glucoamylase in the medium. GroELS and RI were induced at the two cell densities, which corresponded to the following optical densities and specific growth rates: (1) OD_600 _[GroELS] ≈ 3.0 and μ[GroELS] ≈ 0.22 h^-1^; OD_600 _[RI] ≈ 5.0 ± 0.2 and μ[RI] ≈ 0.22 h^-1^; (2) OD_600 _[GroELS] ≈ 9 and μ[GroELS] ≈ 0.15 h^-1^, OD_600_[RI] ≈ 11, μ[RI] ≈ 0.1 h^-1^. With this procedure GroELS synthesis was induced with 0.4 g L^-1 ^of arabinose 2 hours before RI. The cultivation temperature and agitation parameters after chaperon induction were maintained at 37°C, 180 rpm. RI induction was performed with 0.2 mM of IPTG, the temperature was shifted to 22°C, and the culture was continued for 4 h at a shaking speed of 180 rpm. DTT was added as a dry powder at the RI induction point or 2 hours after RI induction to a final concentration of 12 mM.

### Bioreactor processes

Batch and fed-batch cultures were performed in a 15 L Biostat C bioreactor (B. Braun Biotech, Melsungen, Germany) with an initial cultivation volume of 8 litres. The initial culture parameters as follows: the pO_2 _was maintaind at 30% by adapting the stirrer rate and automatic regulation of the air flow (from 0 to 30 liters per min), pH was controlled at 7.0 ± 0.1 by addition of NH_4_OH (25%) or H_3_PO_4 _(2 M). During all bioreactor processes the growth temperature before and after GroELS induction was maintained at 37°C. The temperature in all processes was down-regulated from 37 to 22°C at the RI induction point. A 0.65 M DTT stock solution in MSM was added to the culture 2 hours after RI induction to achieve a final concentration of 12 mM in the cultivation medium. In case of repeated DTT addition a first pulse of 0.65 M DTT stock solution (in MSM) was added to the culture 2 hours after RI induction to achieve final DTT concentration of 12 mM. Additionally two pulses of each 6 mM DTT (final concentration) were added after each of the two following hours.

At the first DTT addition point the air flow was reduced from 30 to 3-4 L min^-1 ^and stirring was manually regulated to maintain an oxygen concentration of about 0% in the culture in the presence of DTT (as in [13]). The glucose feeding rate during the fed-batch cultures was controlled by the Biostat software (version 4.62). All processes were monitored by the MFCS/win 2.0 supervisory system. Exponential feeding profiles were programmed to maintain a specific growth rate of μ ≈ 0.22 h^-1 ^as earlier described [13].

The fed-batch cultivations were started with a volume of 8.0 L of MSM, and containing 8 or 15 g L^-1 ^of glucose, respectively. The fed-batch mode was started after the initial batch cultivation at OD_600 _≈ 9.5 or OD_600 _≈ 18, respectively. GroELS were induced 1 hour before RI induction at OD_600_≈ 12.5-14 or OD_600_≈ 24-26 in both cases respectively at a specific growth rate μ = 0.22 ± 0.02 h^-1 ^under glucose limitation. RI induction during the fed-batch cultures was peformed with 0.2 mM IPTG at OD_600_≈ 18 or OD_600_≈ 38 (μ = 0.22 ± 0.02 h^-1^) and the temperature was shifted to 22°C. The cultures were continued for 5 hours.

The batch cultures were performed in 8 L of MSM medium with 15 g L^-1 ^of glucose. The induction of GroELS at the batch cultivation mode was performed at OD_600_= 3.0 with 0.4 g L^-1 ^of arabinose. RI induction with 0.2 mM IPTG was performed one hour later at an OD_600 _of 6.0 (μ ≈ 0.5 h^-1^) and the temperature was shifted to 22°C. After RI induction the cultures were continued for 5 hours.

### Analytical tools

Cell samples harvested from flask and bioreactor cultivations were resuspended in lysis buffer at the following ratio: 1 g of biomass were resuspended in 10 mL of lysis buffer (50 mM Tris-H_3_PO_4 _pH 8.0, 0.1% Triton X-100, 2 mM EDTA, 1 mM PMSF, 12 mM DTT, 10% propyleneglycol, 0.1 mg mL^-1 ^lysozyme). After 30 min of incubation at +4°C the biomass was sonicated for 60 sec (Vibra cell™, Sonic and Materials Inc., sonotrode 6 mm diameter, amplitude 50%) at 4°C. Soluble and insoluble protein fractions were separated by centrifugation for 30 min, 14000 rpm, 4°C. The total protein fraction represents cellular debris suspension (crude extract) before centrifugation. After centrifugation the insoluble protein pellet was additionally washed and resuspended in the original volume of lysis buffer without lysozyme.

Samples for SDS-PAGE separation were prepared as folllows: 20 μL of protein sample (total soluble, insoluble, protein suspensions), 25 μL of 4 × SDS-PAGE loading buffer (Fermentas), 5 μL of 20 × DTT (Fermentas) and 50 μL of deionized water to obtain a final sample volume of 100 μL. Samples were heated for 15 min at 95°C. 10 μL of sample was applied to each lane of a 10% SDS-PAGE gel.

The amounts in mg of target RI were determined from scanned SDS-PAGE gel images by analysis of the images with TotalLab Quant software (Totallab, Newcaslte, Great Britain). The gels with separated sample proteins were produced for TotalLab quantifications with BSA standards in 3 concentrations on each gel.

The amount of active RI in the soluble protein fraction was determined by an activity assay described by Blackburn et al. [29,30]. One mg of native RI correlates with an activity of about ~100 kU as described by Blackburn et al. [30].

The amount of oxidized/reduced DTT in the cultivation medium was determined by using the Measure-iT™ Thiol Assay kit (Invitrogen) according to the recommendations of the supplier.

## Competing interests

The authors declare that they have no competing interests.

## Authors' contributions

JS designed the experimental setup, performed all cultivation experiments and prepared the manuscript. PN initiated the project, assisted with data analysis and manuscript preparation. Both authors read and approved the final manuscript.

## Supplementary Material

Additional file 1 **SDS-PAGE images of total cell extracts (T), soluble (S), or insoluble (IN) protein fractions normalised to equal cell amounts of *E. coli *ER2566 pET21bRI after 4 hours of batch RI production with addition of 12 mM DTT at the time of RI induction (gels marked with triangles - B, E), 2 hours after RI induction (gels marked with squares - C, F), or no addition of DTT (A, D), respectively**. Batch shake flask cultures were performed in glucose MSM at 37 (gel images: A-C), or 22°C (gel images: D-F). Lane abbreviations: 1T - total protein fraction 10 min before induction, 2 (S), 3 (T) and 4 (IN) - soluble, total and insoluble protein fractions 4 hours after RI induction. Protein size marker: PageRuler™ Protein Ladder Plus (Fermentas). Click here for file

Additional file 2 **SDS-PAGE images of total cell extracts (T), soluble (S), or insoluble (IN) protein fractions normalised to equal cell amounts of *E. coli *ER2566 pET21bRI pKJE7 after 4 hours of batch RI production with addition of 12 mM DTT at the time of RI induction (gels marked with triangles - B, E), 2 hours after RI induction (gels marked with squares - C, F), or no addition of DTT (A, D), respectively**. For explanations see Additional file 1. Click here for file

Additional file 3 **SDS-PAGE images of total cell extracts (T), soluble (S), or insoluble (IN) protein fractions of *E. coli *ER2566 pET21bRI pGro7 normalized to equal cell amounts after 4 hours of batch RI production with addition of 12 mM DTT at the time of RI induction (marked with triangles -B, E, H), 2 hours after RI induction (marked with squares-C, F, I), or no addition of DTT (A, D, G), respectively**. Batch shake flask cultures were performed in glucose MSM at 37 (gel images: A-C), 30 (gel images: D-F), or 22°C (gel images: G-I). For further explanations see Additional file 1. Click here for file

Additional file 4 **SDS-PAGE images of total cell extracts (T), soluble (S), or insoluble (IN) protein fractions from EnBase fed-batch cultures of *E. coli *ER2566 pET21bRI pGro7 normalized to equal cell amounts after 4 hours of RI production without (A) or with addition of 12 mM DTT (B)**. RI was induced at OD_600 _of 11. For further explanations see Additional file 1. Click here for file

Additional file 5 **SDS-PAGE images of total cell extracts (T), soluble (S), or insoluble (IN) protein fractions from batch and fed-batch bioreactor cultures of *E. coli *ER2566 pET21bRI pGro7 normalized to equal cell amounts after 4 hours of RI production without (A) or with addition of DTT (B)**. Gels A and B: protein fractions after RI batch production without (A) and with a single addition of 12 mM DTT (B) added 2 hours after RI induction. Gels C and D: protein fractions after a fed-batch process without (C) and with repeated addition of DTT (D) the first DTT pulse (12 mM ) added 2 hours after RI induction. For further explanations see Additional file 1. Click here for file
