# Supplementary figures and images for: Heterologous production of active ribonuclease inhibitor in Escherichia coli by redox state control and chaperonin coexpression
Source: Microb Cell Fact. 2011 Aug 8;10:65. doi: 10.1186/1475-2859-10-65 (PMC3161860; doi:10.1186/1475-2859-10-65)

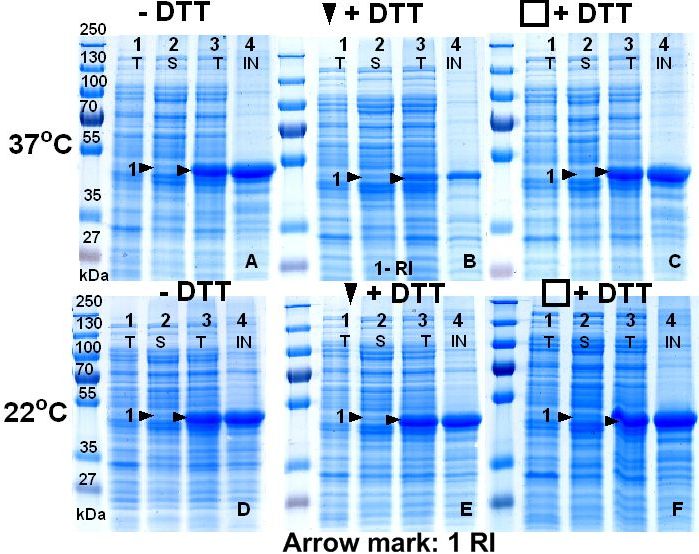

Supplement: Additional file 1 — SDS-PAGE images of total cell extracts (T), soluble (S), or insoluble (IN) protein fractions normalised to equal cell amounts of E. coli ER2566 pET21bRI after 4 hours of batch RI production with addition of 12 mM DTT at the time of RI induction (gels marked with triangles - B, E), 2 hours after RI induction (gels marked with squares - C, F), or no addition of DTT (A, D), respectively. Batch shake flask cultures were performed in glucose MSM at 37 (gel images: A-C), or 22°C (gel images: D-F). Lane abbreviations: 1T - total protein fraction 10 min before induction, 2 (S), 3 (T) and 4 (IN) - soluble, total and insoluble protein fractions 4 hours after RI induction. Protein size marker: PageRuler™ Protein Ladder Plus (Fermentas). [file 1475-2859-10-65-S1.JPEG]

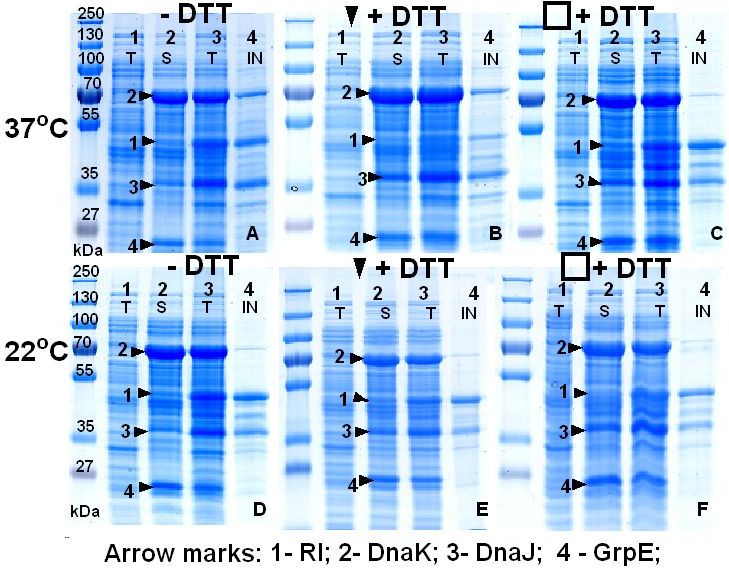

Supplement: Additional file 2 — SDS-PAGE images of total cell extracts (T), soluble (S), or insoluble (IN) protein fractions normalised to equal cell amounts of E. coli ER2566 pET21bRI pKJE7 after 4 hours of batch RI production with addition of 12 mM DTT at the time of RI induction (gels marked with triangles - B, E), 2 hours after RI induction (gels marked with squares - C, F), or no addition of DTT (A, D), respectively. For explanations see Additional file 1. [file 1475-2859-10-65-S2.JPEG]

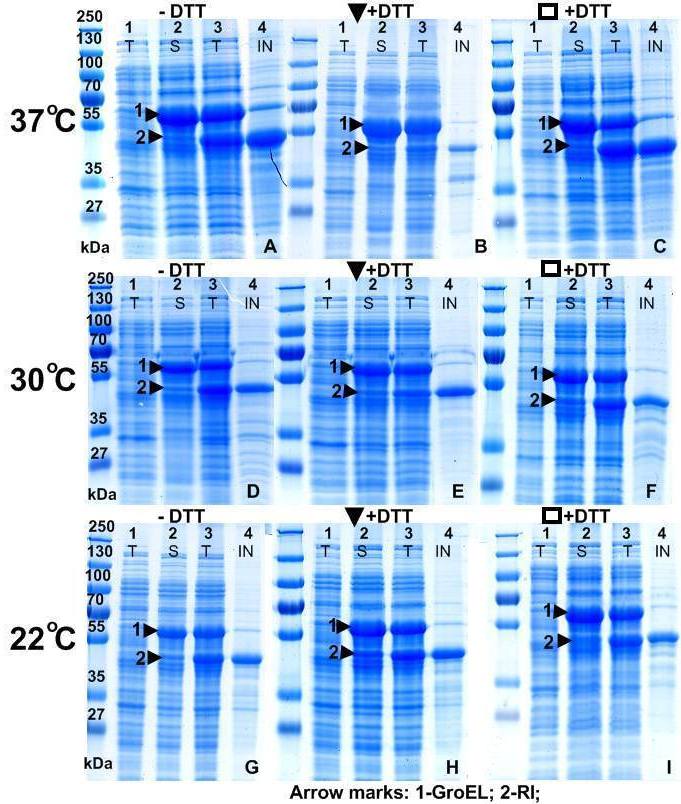

Supplement: Additional file 3 — SDS-PAGE images of total cell extracts (T), soluble (S), or insoluble (IN) protein fractions of E. coli ER2566 pET21bRI pGro7 normalized to equal cell amounts after 4 hours of batch RI production with addition of 12 mM DTT at the time of RI induction (marked with triangles -B, E, H), 2 hours after RI induction (marked with squares-C, F, I), or no addition of DTT (A, D, G), respectively. Batch shake flask cultures were performed in glucose MSM at 37 (gel images: A-C), 30 (gel images: D-F), or 22°C (gel images: G-I). For further explanations see Additional file 1. [file 1475-2859-10-65-S3.JPEG]

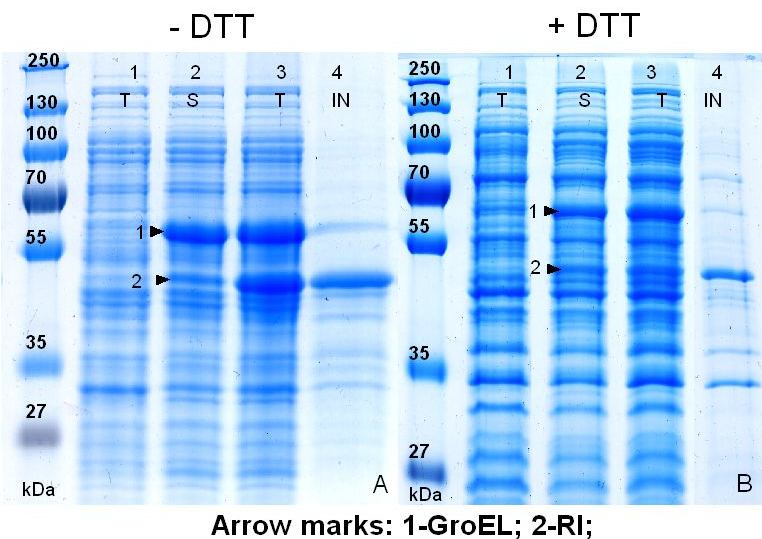

Supplement: Additional file 4 — SDS-PAGE images of total cell extracts (T), soluble (S), or insoluble (IN) protein fractions from EnBase fed-batch cultures of E. coli ER2566 pET21bRI pGro7 normalized to equal cell amounts after 4 hours of RI production without (A) or with addition of 12 mM DTT (B). RI was induced at OD600 of 11. For further explanations see Additional file 1. [file 1475-2859-10-65-S4.JPEG]

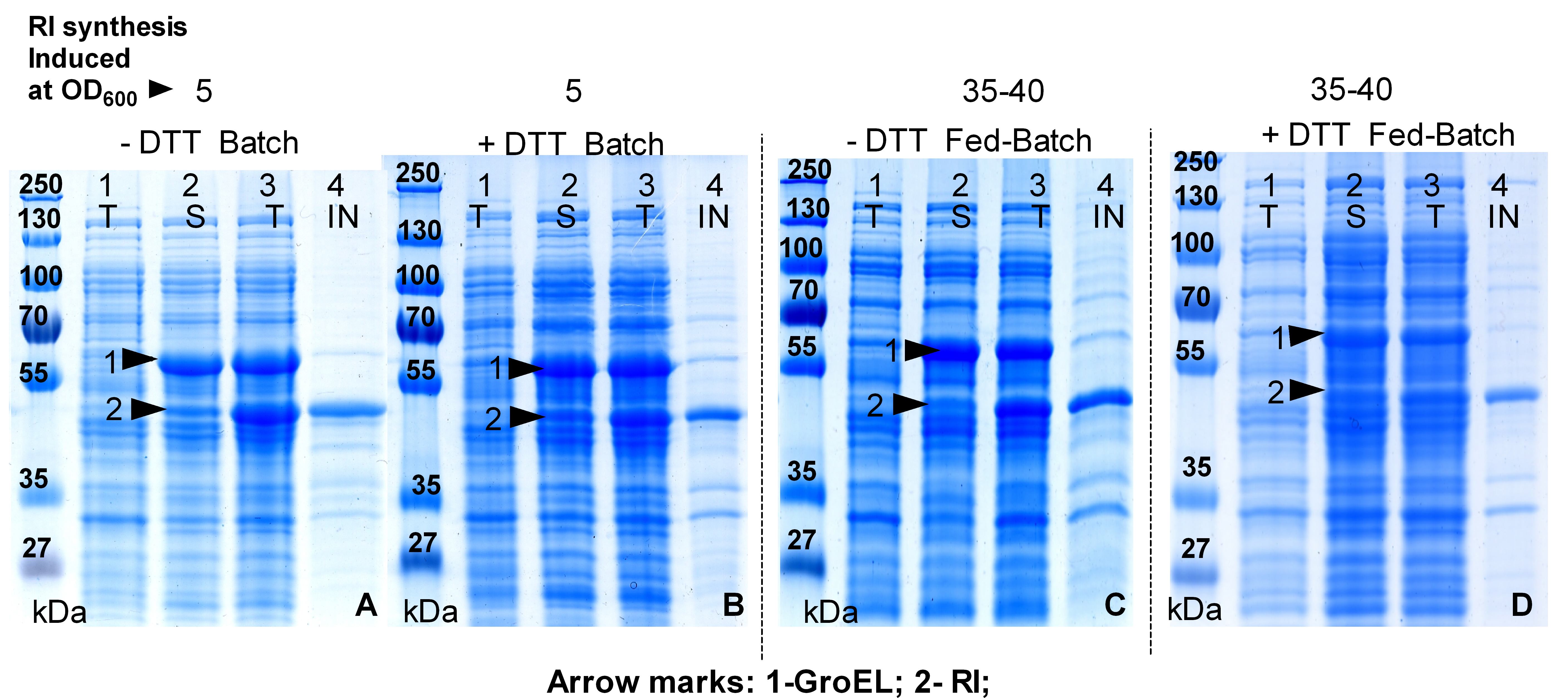

Supplement: Additional file 5 — SDS-PAGE images of total cell extracts (T), soluble (S), or insoluble (IN) protein fractions from batch and fed-batch bioreactor cultures of E. coli ER2566 pET21bRI pGro7 normalized to equal cell amounts after 4 hours of RI production without (A) or with addition of DTT (B). Gels A and B: protein fractions after RI batch production without (A) and with a single addition of 12 mM DTT (B) added 2 hours after RI induction. Gels C and D: protein fractions after a fed-batch process without (C) and with repeated addition of DTT (D) the first DTT pulse (12 mM ) added 2 hours after RI induction. For further explanations see Additional file 1. [file 1475-2859-10-65-S5.JPEG]
